# Overexpression of a ceramide synthase gene,*GhCS1*, inhibits fiber cell initiation and elongation by promoting the synthesis of ceramides containing dihydroxy LCB and VLCFA

**DOI:** 10.3389/fpls.2022.1000348

**Published:** 2022-09-02

**Authors:** Guiming Li, Qiaoling Wang, Qian Meng, Guanhua Wang, Fan Xu, Qian Chen, Fang Liu, Yulin Hu, Ming Luo

**Affiliations:** ^1^Key Laboratory of Biotechnology and Crop Quality Improvement, Ministry of Agriculture/Biotechnology Research Center, Southwest University, Chongqing, China; ^2^Key Laboratory of Horticulture Science for Southern Mountains Regions of Ministry of Education, College of Horticulture and Landscape Architecture, Southwest University, Chongqing, China; ^3^Academy of Agricultural Sciences of Southwest University, State Cultivation Base of Crop Stress Biology for Southern Mountainous Land of Southwest University, Chongqing, China

**Keywords:** cotton, fiber cell, sphingolipids, ceramide synthase, S1P, ceramide, ROS

## Abstract

Cotton is an important natural fiber crop worldwide. Cotton fiber cell is regarded as an ideal material for studying the growth and development of plant cells. Sphingolipids are important components of biomembrane and bioactive molecules which participate in many processes such as plant growth, development regulation, stimulus sensing, and stress response. However, the functions of sphingolipids in the cotton fiber development are still unclear. In the present study, we identified a cotton ceramide synthase gene, *GhCS1*, which is predominantly expressed in fiber cell. The GhCS1 is located in the endoplasmic reticulum and has the conserved domains of ceramide synthase. Overexpression of *GhCS1* gene inhibited both vegetative and reproductive growth in cotton. Importantly, the fiber cell initiation and elongation were severely inhibited when compared with control. Comparison of the sphingolipid profile in the 0-DPA (days past anthesis) ovule (with fiber cell) between control and transgenic cotton plants showed that the content of sphingosines (Sph) decreased significantly in transgenic ovules, whereas the content of phyto-sphingosines (Phyto-Sph) had no change. Meanwhile, the content of ceramide containing Sph and very-long-chain fatty acid (VLCFA) increased significantly in transgenic ovules, while ceramide containing Phyto-Sph and long-chain fatty acids (LCFA)/VLCFA significantly decreased. These results indicated that *GhCS1* was a functional ceramide synthase, which preferentially used Sph and VLCFA as substrates and was different from the *Arabidopsis* ceramide synthase AtLOH1*/*AtLOH3, which preferentially used Phyto-Sph and VLCFA as substrates, and also different from AtLOH2, which preferentially used Sph and LCFA as substrates. It is suggested that *GhCS1* might be a new ceramide synthase gene in the plant, play some roles in the development of fiber cells and cotton plants.

## Introduction

Cotton is an important natural fiber crop in the world. Cotton fiber is a single-celled trichome fiber formed from the ovule outer integument epidermal cells through initiation, elongation, and secondary cell wall (SCW) deposition. Fiber cells are not broken by cell division during the development process, and cell elongation and secondary wall synthesis last for a long time (about 20 days). Therefore, fiber cells are regarded as ideal materials for studying plant cell development (Qin and Zhu, 2011; Haigler et al., 2012). At the same time, the elongation and SCW deposition of fiber cells directly affect the length and strength of mature fibers and other important quality traits. Therefore, fiber development is a focus in cotton research (Kim and Triplett, 2001; Deng et al., 2016; Niu et al., 2019; Huang et al., 2021). In recent years, due to the publication of several cotton genome sequences, the study on cotton fiber development has made great progress (Zhang et al., 2011; Wang et al., 2019; Huang et al., 2021). However, the regulatory mechanism of fiber cell development is still unclear to date.

Sphingolipids are not only important components of membrane, but also vital bioactive molecules, which participate in many physiological and biochemical processes such as membrane structure, signal transduction, immunity, membrane trafficking, cell polarity, and growth (Liu et al., 2021). Previous studies have shown that sphingolipids account for 40% of plant cytoplasmic membrane lipids (Cacas et al., 2016). Sphingolipid synthesis inhibitor Fumonisin B1 (FB1) causes auxin carrier protein delocalization by inhibiting ceramide synthesis (Markham et al., 2011). Blocking the glucosylceramides (GluCer) synthesis by application of PDMP (D, L-threo-1-phenyl-2-decanoylamino-3-morpholino-1-propanol) led to changes in Golgi morphology and inhibits the Golgi-dependent protein secretion pathway (Melser et al., 2010). Sphingolipids are abundant in the microdomain of membrane, thus limiting protein diffusion. For example, the changes of membrane sphingolipids affect the aggregation of plasmodesmata (PD) proteins and ultimately affect PD function (Huang et al., 2019; Yan et al., 2019; Liu et al., 2020). Similarly, increasing the saturation of long-chain bases (LCBs) in sphingolipid molecules declined the tolerance to low temperature in plants, since the unsaturation of sphingolipids might be associated to the fluidity of membranes and the maintenance of H^+^-ATPase function in plasma membrane. In addition, low temperature induces the increase of glycosyl inositol phosphoceramides (GIPCs) and the decrease of glycosylceramides (GluCers) in *Arabidopsis* (Chen et al., 2012; Nagano et al., 2014). On the cell membrane, GIPC could bind to fungal toxin NLP to trigger signal transduction, and combine with Na^+^ to transmit salt stress signal and finally initiate the inflow of Ca^2+^ (Lenarčič et al., 2017; Jiang et al., 2019). Sphingosine-1-phosphate (S1P) affects the stomatal aperture by affecting ABA-mediated signal pathway (Coursol et al., 2003). Drought-induced S1P elevation in the leaf and the exogenous application of S1P stimulated Ca^2+^spike and stomatal closure (Ng et al., 2001; Puli et al., 2016). These studies revealed that sphingolipids play roles in the growth and development of plant although most studies focused on biotic and abiotic stress.

Based on metabolome and transcriptome analysis, we have showed that sphingolipids play some important roles in fiber cell development as follows: ceramide that contains phyto-sphingosine (tri-hydroxyl LCB) and saturated VLCFA enriched in the elongation stage of fibers (Chen et al., 2021); GluCer and GIPC were significantly reduced in the 0-DPA (Day Post Anthesis) ovules of two *lintless-fizzless* mutants, *Xuzhou 142* and *Xinxiang Xiaoji*, while four ceramide molecules (d18:1/22:0 and d18:1/24:0, t18:0/22:0, and t18:0/h22:1) were significantly increased in the mutants (Wang et al., 2021b). Fumonisins (FB1) is a specific inhibitor of ceramide synthase (Abbas et al., 1994; Luttgeharm et al., 2016). Exogenous application of FB1 significantly reduced the contents of GluCer, GIPC, and Cer (ceramides) in fiber cells, while significantly increased the contents of Sph and S1P, and then inhibited the growth of fibers (Wang et al., 2020). Using another sphingolipid synthesis inhibitor, myriocin (specifically inhibiting serine palmitate acyltransferase) could inhibit the synthesis of sphingolipids and cotton embryo growth (Wang et al., 2021a). However, these studies were focused on the biochemical level. There is no genetic evidence to illuminate the role of sphingolipids in the growth and development of cotton fiber cell.

Ceramide synthesis mediated by ceramide synthase is the center of the sphingolipid synthesis pathway. Ceramide synthase catalyzes the combination sphingosine and FA (VLCFA or LCFA) to form ceramide. The ceramide can further generate two types of sphingolipids, GluCer and GIPC, and then phosphorylate to form Cer-1-P, and modify in FA and LCB chains to form different sphingolipid molecules (Liu et al., 2021). The *Arabidopsis* genome contains three genes that encoding ceramide synthase, which are LONGEVITY ASSOCIATION GENE 1 homolog, *LOH1* (At3g25540), *LOH2* (At3g19260), and *LOH3* (At1g13580; Markham et al., 2011; Ternes et al., 2011). LOH1 and LOH3 have about 80% amino acid sequence identity and mainly catalyze the amidation of CoA esters of tri-hydroxyl LCB and VLCFA. LOH2 amino acid sequence is similar as LOH1 and LOH3, and mainly catalyzes the condensation of di-hydroxyl LCB and C16 fatty acyl CoA (Chen et al., 2008; Markham et al., 2011). The ceramide synthesized by LOH1 and LOH3 is enriched in GIPC molecules while the ceramide synthesized by LOH2 is enriched in GluCer (Chen et al., 2008; Ternes et al., 2011). Overexpression of *LOH1* and *LOH3* promoted cell division and plant growth. On the contrary, the *loh1loh3* double mutant was sterility indicating ceramide synthase was important for plant development. However, *LOH2* overexpression led to the increase of ceramide containing C16 fatty acid and di-hydroxyl Sph, and induced programmed cell death and salicylic acid accumulation, resulting in smaller and shorter plants (Luttgeharm et al., 2015). Plants overexpressing *LOH2* and *LOH3* have increased resistance to FB1 while plants overexpressing *LOH1* have not increased resistance (Luttgeharm et al., 2015). These results indicate that different ceramide synthases have different substrate specificity and have different functions in regulating plant growth and development.

There are 10 homologs of ceramide synthase in the upland cotton (*Gossypium hirsutum* L.) genome. However, the function of these genes in cotton plant growth, especially, in cotton fiber development is still unclear. Through analyzing the sequence of ceramide synthase in the upland cotton genome and upregulating *GhCS1* expression in transgenic cotton plants, we clarified that *GhCS1*, which was preferentially expressed in fiber cells, was a new ceramide synthase in plants with special substrate preference and function, and played important roles in cotton fiber initiation and elongation. Our study laid a foundation for further revealing the role of sphingolipids in cotton growth and development.

## Materials and methods

### Plant materials and growth conditions

The cotton plant used in this study was upland cotton (*Gossypium hirsutum* L.) cv. Jimian14, which was kindly provided by Professor Zhiying Ma (Hebei Agricultural University, China). Transgenic cotton plants were grown in the greenhouse under natural and additional artificial light (14 h light photoperiod at 150 μmol m^−2^ s^−1^) at 28°C–34°C during the day and 24°C–27°C at night.

### RNA extraction and QRT-PCR

Total RNA was isolated from leaves, roots, stems, petals, pistils, stamens, flowers at 0 days post-anthesis (DPA), 0–4 DPA ovules, and 6–20 DPA fibers using a Plant Total RNA Extraction kit (Tiangen, China) according to the manufacturer’s instructions, and converted to cDNA using a Reverse Transcription Kit with Genomic DNA Remover (Takara, Japan) according to the manufacturer’s instructions. QRT-PCR analysis was performed using Novostar-SYBR qPCR Supermix (Novoprotein, Shanghai, China) as follows: 93°C for 3 min followed by 40 cycles of 95°C for 15 s, 56°C for 30 s, and 72°C for 30 min. Cotton HISTONE3 (GenBank accession no. AF024716) was used to normalize the data. The primers for HISTONE3 were HIS3-P1 (5′-GAAGCCTCATCGATACCGTC-3′) and HIS3-P2 (5′-CTACCACTACCATCA TGGC-3′). Three biological replications were performed.

### Vector construction and plant transformation

Plant expression vector (pBI121-GN-AK) was constructed according to a previously described method (Luo et al., 2007). Briefly, to construct the overexpression *GhCS1* cassette, the *GhCS1* cDNA fragment in cloning vector was digested by KpnI and EcoRV, and then inserted into pBI121-GN vector at KpnI and EcoRV sites. The expression vector was transferred into Agrobacterium tumefaciens strains (LBA4404) for genetic transformation of cotton. The detailed steps, please refer to the method of Luo et al. (2007), with slightly modified.

### Subcellular localization of GhCS1

The coding sequences of *GhCS1* were amplified and cloned into the binary vector pLGN-eYFP to construct the GhCS1-eYFP vectors. The constructed vectors were transformed into *Agrobacterium tumefaciens* strain GV3101, and then transiently transfected in tobacco leaves as previously reported (Wroblewski et al., 2005). Transfected tobacco plants were kept in dark for 16 h and then moved to light conditions for 48 h. The fluorescence signals were detected using confocal laser scanning microscopy (Leica, Wetzlar SP8, Germany). HDEL was used as endoplasmic reticulum markers. All experiments were performed with three independent biological replicates.

### *In vitro* ovule culture and fiber length measurement

For *in vitro* ovule cultures, cotton ovules were collected at 2-DPA, sterilized in a 3‰ H_2_O_2_ solution, and cultured in Beasley and Ting’s medium (Beasley, 1973) at 32°C in the dark for 5, 10, and 15 days. To test the fiber length, at least 10 ovules were used. The cultured ovules were immersed in 30% glacial acetic acid and were heated in boiling water until the fibers dispersed. The ovules were placed on a slide and were rinsed with water to straighten the fibers, which were then measured. Three biological replicates were performed for this assay.

### 2′,7′-dichlorodihydrofluorescein diacetate staining and imaging

To examine the ROS amounts in transgenic cotton, cotton ovules are detached carefully from bolls, washed with sterile water to remove possible ROS released by cutting, and incubated for 30 min in dark at 25°C in 10 μmol/L 2′,7′-dichlorodihydrofluorescein diacetate (2,7-DCFDA) dissolved in DMSO. Then washed with sterile water and left for 10 min at 25°C before imaging. Fluorescence images were obtained with a Leica, Wetzlar SP8 confocal spectral microscope. Dye excitation was at 488 nm; emitted light was detected at 522 nm. Images were processed with Leica Confocal Software (Leica, Wetzlar SP8, Germany).

### GUS staining

Leaves from transgenic plants were examined for the presence of the CaMV 35S promoter::GUS::Nos gene using a histochemical assay for GUS activity. The plant leaves were incubated overnight at 37°C in the staining solution (1.5 mg/mL 5-Bromo-4-chloro-3- indolyl-beta-D-glucuronide, 50 mM sodium phosphate buffer, pH 7.0, 0.1% (v/v) Triton X100, 0.5 mM potassium ferricyanide; and 0.5 mM potassium ferrocyanide). After incubation, the plant leaves were faded in a clearing solution of 8:3:1 (w:v:v) chloral hydrate:distilled water:glycerol. Then, the samples were observed by stereoscope (SteREO Discovery. V20, Zeiss, Gottingen, Germany).

### Iodine-potassium iodide staining

Taking a glass slide, add 1–2 drops of Iodine-potassium iodide (I_2_-KI) staining solution (6.5 g iodine and 17.5 g KI dissolved in 100 ml water), gently shaking the pollen into the staining solution, covering with a cover glass, and leaving it at room temperature for 3–5 min and then observing under the microscope.

### Lipid extraction and lipidomics

After fiber sample collection, lipid extraction and lipidomic analysis were performed by LipidAll Technologies Company Limited,^1^ as described previously (Welti et al., 2002; Markham and Jaworski, 2007; Huang et al., 2019; Wang et al., 2020). Briefly, the analyses were conducted using an Exion ultra-performance liquid chromatography (UPLC; AB Sciex, CA, United States) coupled with a Sciex QTRAP 6500 PLUS (AB Sciex, CA, United States). The lipids were separated using a Phenomenex Luna 3 μm silica column (Phenomenex, CA, United States; internal diameter: 150 mm × 2.0 mm) under the following conditions: mobile phase A (chloroform:methanol:ammonium hydroxide, 89.5:10:0.5) and mobile phase B (chloroform:methanol:ammonium hydroxide:water, 55:39:0.5:5.5). The gradient began with 95% of mobile phase A for 5 min and was followed by a linear reduction to 60% mobile phase A over 7 min. The gradient was held for 4 min, and mobile phase A was then further reduced to 30% and was held for 15 min. MRM transitions were constructed for a comparative analysis of the various sphingolipids. The individual sphingolipid classes were quantified by referencing spiked internal standards, namely Cer d18:1/17:0, GluCer d18:1/12:0, d17:1-S1P, D-ribo-phyto-sphingosine C17, and d17:1-Sph from Avanti Polar Lipids (Alabaster, AL, United States) and GM1 d18:1/18:0-d3 from Matreya LLC. (State College, PA, United States).

### Scanning electron microscope

The 0-DPA ovaries of transgenic plants and wild-type plants were collected. The fresh ovules taken from ovaries were observed using a scanning electron microscope (SEM; SU 3500, Hitachi, Tokyo, Japan).

### Statistical data analysis

Data were presented as mean ± SD. Statistical data analysis was performed by the one-tailed Student’s *t*-test. ^*^, ^**^, and ^***^ indicate significant differences at *p* < 0.05, <0.01, and <0.001, respectively.

## Results

### Characters of upland cotton *GhCS* genes

**Figure 1:**
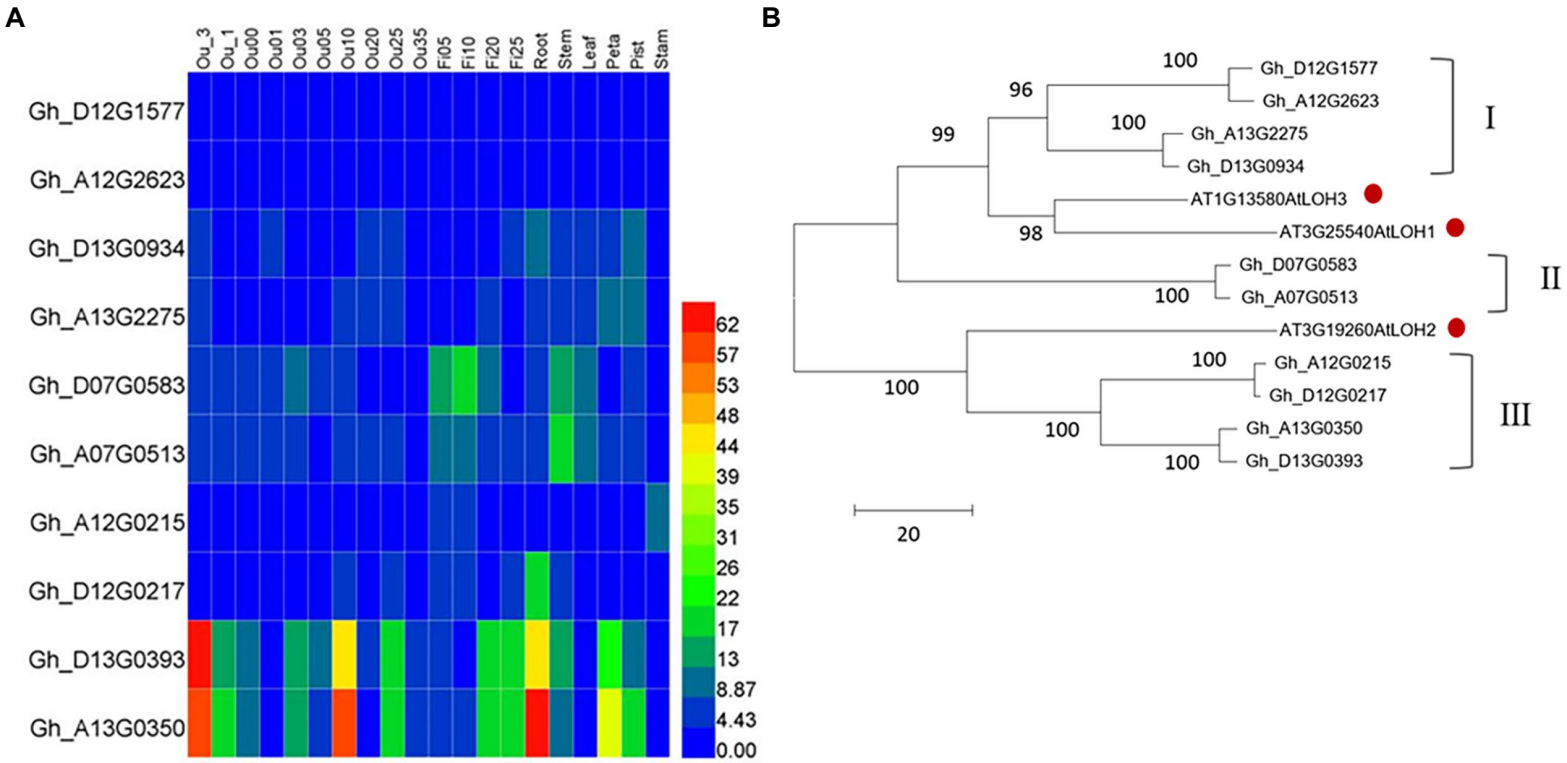
Characters of upland cotton *GhCS* genes. **(A)** The expression pattern of genes encoding ceramide synthase in upland cotton. **(B)** The phylogenetic relationship of cotton ceramide synthase with *Arabidopsis thaliana* homologs. The red dot indicated the *Arabidopsis* ceramide synthase. Ou_1 and Ou_3: the ovule of 1 and 3 days (s) before anthesis; Ou00: the ovule (with fiber cells) on the day of anthesis; Ou01 and Ou3: the ovule (with fiber cells) of 1 and 3 days (s) post anthesis, respectively; Fi05 to Fi25: the fiber cells of 5 to 25 days post anthesis; Ou05 to Ou35: the ovule (without fiber cells) of 5 to 35 day (s) post anthesis.

There are three ceramide synthase (CS) genes in the *Arabidopsis thaliana* genome, which are At3g25540 (*AtLOH1*), At3g19260 (*AtLOH2*), and At1g13580 (*AtLOH3*). Using their protein sequence as a probe, we blasted in the upland cotton database FGD^2^ and obtained 10 genes with highly homology, namely Gh_D13G0934, Gh_A13G2275, Gh_A12G2623, Gh_D12G1577, Gh_D07G0583, Gh_A07G0513, Gh_A12G0215, Gh_D13G0393, Gh_D12G0217, and Gh_A13G0350. The basic information of these 10 genes is shown in Table 1. By screening the transcriptome of various samples from cotton, we found that the overall expression levels of Gh_D13G0393 and Gh_13G0350 were the highest, while that of Gh_A12G2623 and Gh_D12G1577 were the lowest among the 10 *GhCS* genes. Furthermore, Gh_D13G0393 and Gh_A13G0350 were highly expressed in the differentiation and initiation stages of fiber cells (−3 ~ 0 DPA), secondary wall deposition stage (20 ~ 25 DPA), 10-DPA ovules, and roots. Gh_D07G0583 and Gh_A07G0513 highly expressed in fibers and stems (Figure 1A). Intriguingly, the expression peak of Gh_D07G0583 was the rapid fiber elongation period (5–10 DPA), suggesting that it might play some roles in cotton fiber elongation.

**Table 1:** Ten identified ceramide synthase genes (*GhCSs*) in upland cotton genome.

Locus name	Chr^a^	Location coordinates	ORF length (bp)		Protein		Length(AA)	mass(Da)	PI
Gh_D13G0934	A13	4,632,743–4,643,090	921	307	36,234	7.42
Gh_A13G2275	A13	70,800–74,443	963	321	37,647	8.72
Gh_A12G2623	A12	57,007–60,892	948	316	37,090	8.78
Gh_D12G1577	D12	46,856,738–46,864,694	1803	614	69,768	6.60
Gh_D07G0583	D07	6,645,792–6,649,427	924	308	36,076	8.19
Gh_A07G0513	A07	6,705,558–6,709,212	924	308	36,248	7.87
Gh_A12G0215	A12	3,261,197–3,265,193	873	291	34,213	7.29
Gh_D13G0393	D13	4,545,850–4,551,778	870	290	34,024	7.98
Gh_D12G0217	D12	3,064,350–3,068,922	873	291	34,173	7.30
Gh_A13G0350	A13	5,245,629–5,251,118	870	290	34,014	7.97

a Chromosomal localization of the GhCS genes.

Phylogenetic tree of cotton and *Arabidopsis* ceramide synthases (Figure 1B) showed that the 13 enzymes were clustered into two main clades. The larger branch contained six GhCSs and two AtLOHs. Among them, the six GhCSs were obviously divided into two subgroups (I and II). Subgroup I contained four GhCSs and subgroup II contained only two GhCSs. The GhCSs of subgroups I and II were closely related to the AtLOH1 and AtLOH3 of *Arabidopsis*. Another branch contained *AtLOH2* and 4 GhCSs (subgroup III). The result suggested that Gh_D07G0583 and Gh_A07G0513 might have special sequence.

### Identification of *GhCS1*

**Figure 2:**
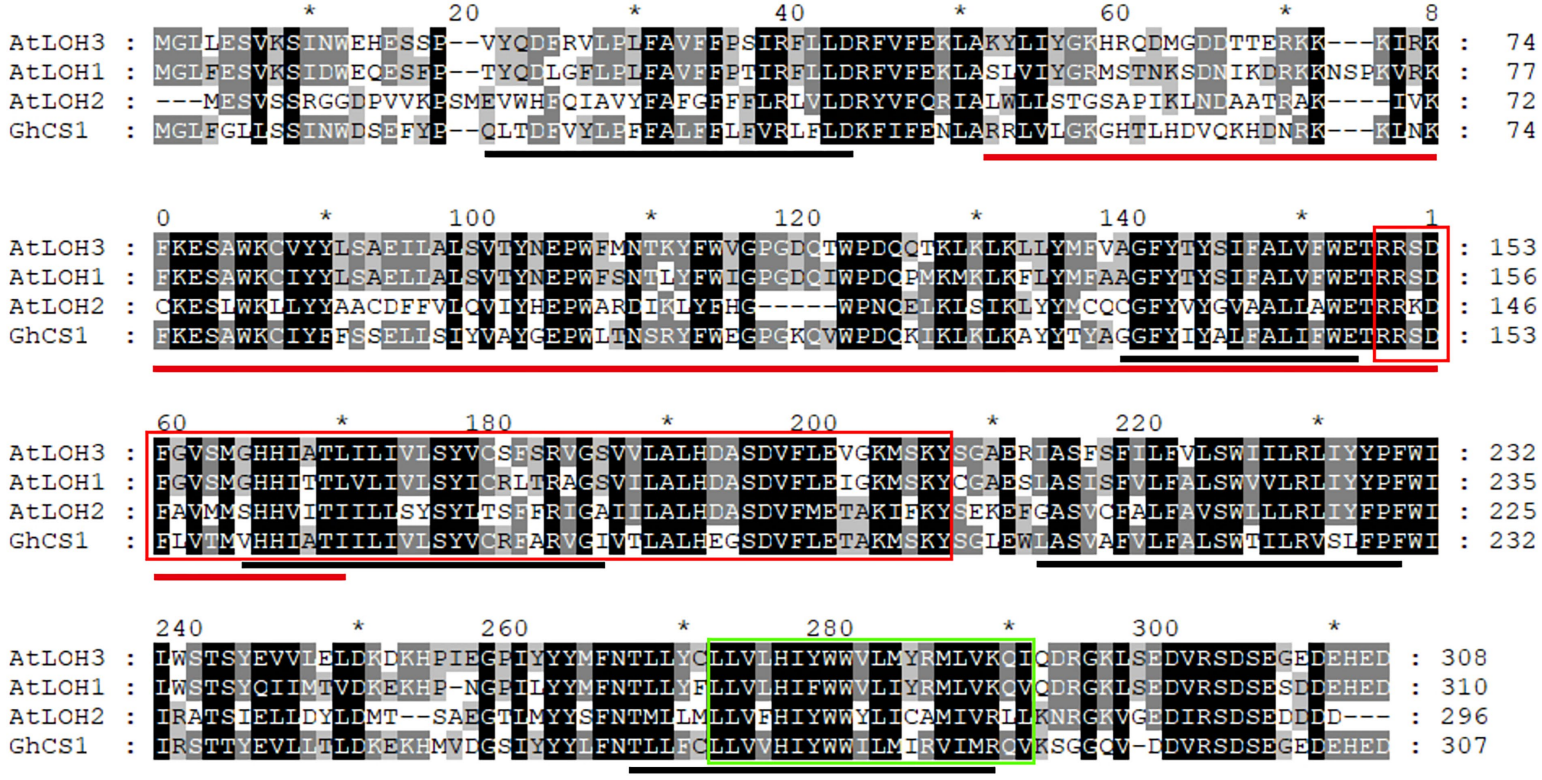
Alignment of GhCS1 with three *Arabidopsis* ceramide synthase. AtLOH1 ~ 3: *Arabidopsis* ceramide synthase 1 ~ 3. The red line shows the conserved domain of TLC; the black line shows the five transmembrane regions; the red box represents the conserved domain of LAG1 (LONGEVITY ASSURANCE GENE 1); the green box represents the conserved domain of glycerol-3-phosphate acyltransferase.

According to the transcriptome analysis, *Gh_D07G0583* and *Gh_A07G0513* genes were highly expressed in fiber elongation, and especially, the expression of *Gh_D07G0583* was the highest during rapid elongation stage of fiber (Figure 1A). It was speculated that this gene had some roles in fiber cell elongation. Using the cDNA from upland cotton Jimian14 10-DPA fiber as a template, we cloned *Gh_D07G0583* gene. The cDNA sequence of this gene is 1,211 bp, including a 924 bp ORF encoding 307 amino acid residues, with a molecular weight of 36 KD and an isoelectric point of 8.190. In order to clarify the genomic structure of this gene, its genomic DNA was amplified by using the same primers, and the resulting sequence was 3,647 bp. Compared with the cDNA sequence, the genomic sequence of the gene contained 6 exons and 5 introns. This gene is located on chromosome 7 of D sub-genome and we named it *GhCS1*. The GhCS1 contains five transmembrane domains (Figure 2). Based on the multi-sequence alignment, the GhCS1 has the same conserved domain TLC as the CS of other species, which contains a LAG1 (LONGEVITY ASSURANCE GENE 1) conserved domain and a glycerol-3-phosphate acyltransferase domain that are necessary for ceramide synthesis dependent on acyl-coenzyme A.

### The expression pattern and subcellular location of GhCS1

**Figure 3:**
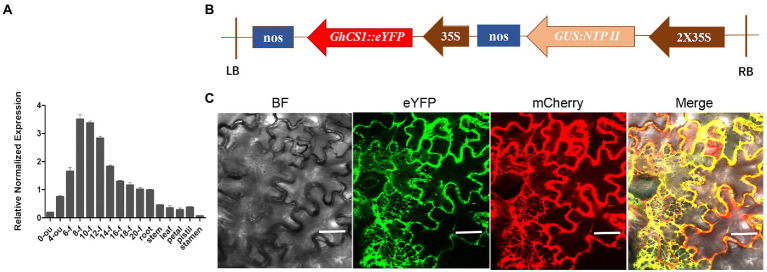
Expression pattern of *GhCS1* and GhCS1 subcellular localization. **(A)** The expression pattern of *GhCS1*. 0-ou and 4-ou: 0 DPA and 4 DPA ovules (with fiber cells); 6-f~20-f: The fiber cells of 6 to 20 DPA. Error bars represent the standard deviation (SD) of three independent replicate experiments. **(B)** The diagram of plant expression vector of *GhCS1*::eYFP fusion gene; **(C)** The subcellular location GhCS1::eYFP protein. BF: bright field; mCherry: HDEL::mCherry (HDEL is a marker protein of endoplasmic reticulum); eYFP: GhCS1::eYFP; Bar = 40 μm.

To understand the expression characteristics of *GhCS1* gene in cotton plant, we used real-time quantitative PCR to detect the expression of *GhCS1* gene in different tissues and organs of upland cotton Jimian14, and also at different stages of ovule and fiber development (Figure 3A). The results showed that the expression of *GhCS1* gene was the highest in fiber, followed by ovule and root, and low in flower, stem, and leaf. During ovule and fiber development, the expression level of *GhCS1* gene gradually increased from the day of anthesis, peaked at 8 to 12 days post anthesis(DPA), and then gradually decreased. Given that 8–12 DPA is the rapid elongation period of fiber cells, these results suggested that *GhCS1* might be involved in the rapid elongation of cotton fiber, and might play a role in cotton fiber elongation. In order to clarify the subcellular localization of GhCS1, a plant expression vector (Figure 3B) with the constitutive promoter CaMV 35S controlling the GhCS1::eYFP fusion gene was constructed and injected into tobacco leaves for transient expression. By laser scanning confocal fluorescence microscopy, we observed that eYFP signal overlapped with the signal of HDEL-mCherry, which fused with the endoplasmic reticulum marker protein HDEL (Figure 3C), indicating that GhCS1 protein was localized in the endoplasmic reticulum.

### Overexpression of *GhCS1* seriously affects the development of cotton reproductive organs

**Figure 4:**
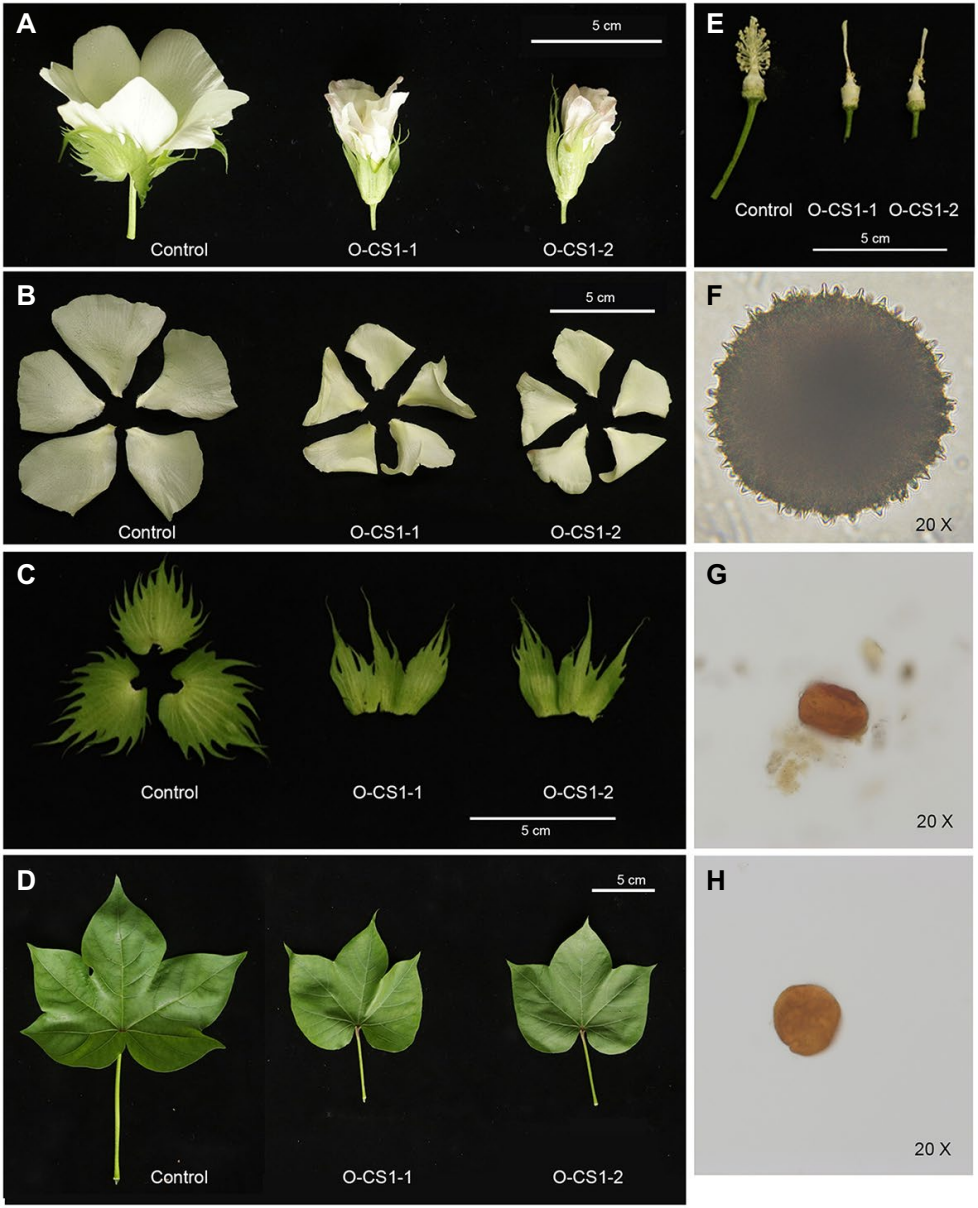
Phenotypes of *GhCS1*-overexpression lines. **(A)** The 0-DPA flowers from transgenic cotton plant and control plant. **(B)** The petals of transgenic cotton plant and control plant. **(C)** The sepals from transgenic cotton plant and control plant. **(D)** The leaves from transgenic cotton plant and control plant. **(E)** The stamen and pistil of control plant and transgenic cotton plants. **(F)** Control plant pollen. **(G)** The pollen of O-CS1-1. **(H)** The pollen of O-CS1-2. O-CS1-1 and O-CS1-2: Overexpressing *GhCS1* transgenic cotton lines 1# and 2#.

Through Agrobacterium-mediated genetic transformation of cotton, we obtained four transgenic cotton plants with Kan resistance and these lines were further confirmed by histochemical staining and PCR (Supplementary Figure S1). To understand the expression change of *GhCS1* in transgenic plants, we detected the expression level of *GhCS1* in control and transgenic cotton plants by qRT-PCR. The result showed that the GhCS1 expression was significantly increased in the transgenic cotton plants (Supplementary Figure S1), indicating that we obtained *GhCS1*-overexpression cotton plants. Compared with the control plants, the *GhCS1*-overexpression lines exhibited shorter and compact phenotypes with significantly smaller flowers, partially curled petal, smaller bracts and connected bases, smaller leaf area, and sharper leaf shape (Figures 4A–D). The filaments of *GhCS1*-overexpression lines were extremely very short, and there were no normal pollen and only a few abortive pollens (Figures 4E–H). These results showed that overexpression of *GhCS1* gene inhibited the vegetative growth and reproductive organ development of cotton plants to a certain extent.

### Overexpression of *GhCS1* inhibited fiber cell initiation

**Figure 5:**
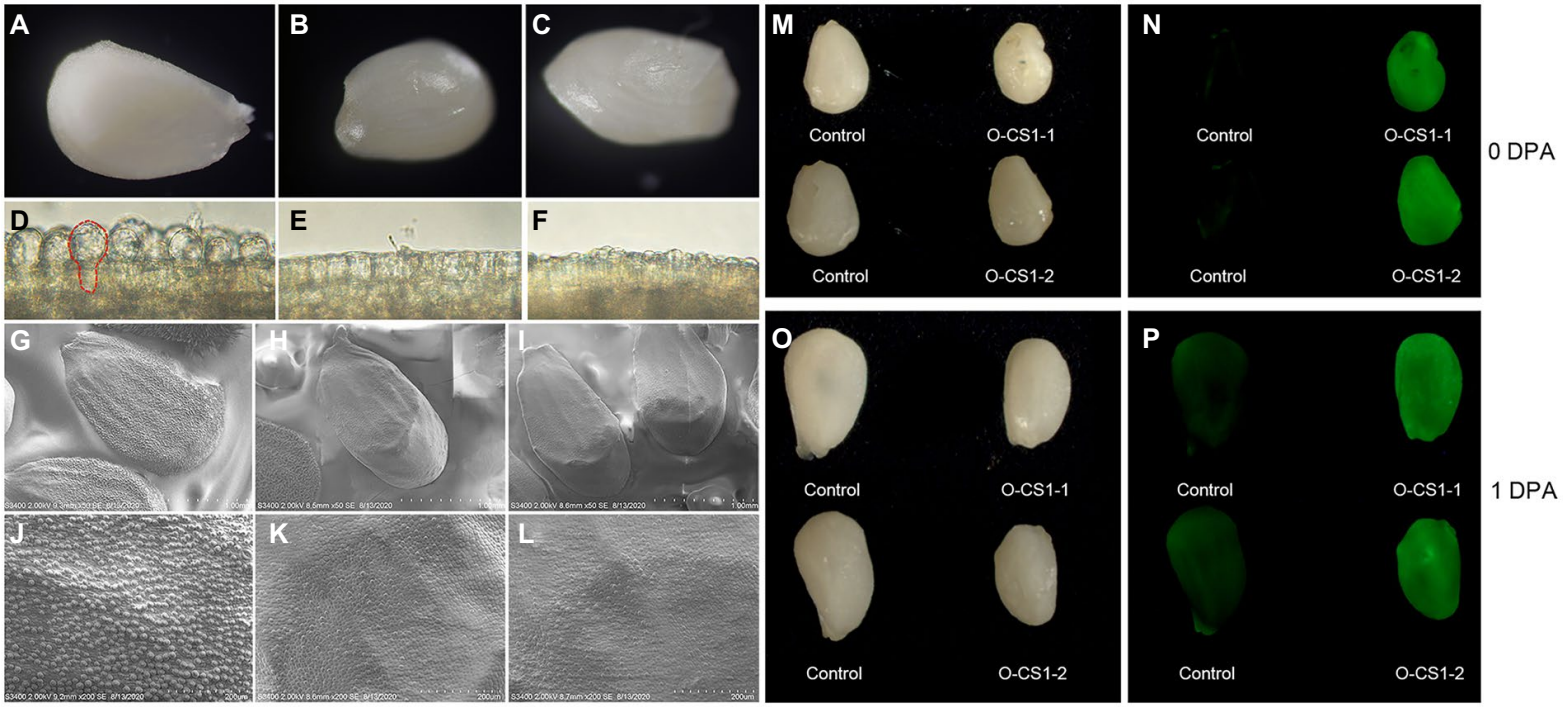
The fiber cell initiation and ROS accumulation in 0-DPA and 1-DPA ovules. **(A–C)** The entire surface of the control and transgenic lines O-CS1-1, O-CS1-2 0-DPA ovules, respectively; **(D–F)** respectively an enlarged area of the ovule shown in **(A–C)**; **(G–L)** The 0-DPA ovule of control and transgenic lines O-CS1-1, O-CS1-2 photos taken by SEM, **(G–I)** are the entire surface of the ovule, and **(J–L)** are partial enlargements of panels **(G–I)**, respectively. **(M,N)** The 0-DPA ovules of control and transgenic plants under light and UV light. **(O,P)** The 1-DPA ovules of control and transgenic plants under light and UV light. O-CS1-1 and O-CS1-2: Transgenic cotton lines 1# and 2# overexpressing GhCS1.

In order to clarify the effect of overexpression of *GhCS1* gene on cotton fiber development, we observed the 0-DPA ovule of overexpressing *GhCS1* transgenic cotton by stereoscope and microscope. The results showed that there were obvious fiber protrusions on the surface of the control 0-DPA ovules (Figures 5A,D), while there were almost no fiber protrusions on the surface of the transgenic 0-DPA ovules (Figures 5B,C,E,F). We then observed the initiation of fiber by the scanning electron microscope. The results showed that there were a large number of fiber protrusions on the surface of the control 0-DPA ovules (Figures 5G,J), while there were only a small number of fiber on the surface of the transgenic 0-DPA ovules, and the initial fiber cells were small (Figures 5H,I,K,L). These results suggest that overexpressing *GhCS1* inhibited fiber initiation. Overexpressing *AtLOH2* also inhibits plant growth and promotes programmed cell death(PCD) in *Arabidopsis* (Luttgeharm et al., 2015). Given that PCD is often accompanied by the accumulation of reactive oxygen species (ROS), we detect the content of ROS in the ovules of *GhCS1* transgenic cotton using DCFH-DA fluorescent dye. The results showed that the content of ROS in transgenic 0-DPA and 1-DPA ovules was significantly higher than that of control (Figures 5M–P). These results showed that overexpression of *GhCS1* gene led to an increase in the content of ROS during fiber initiation.

### Fiber cell elongation was suppressed in *GhCS1*-overexpression lines

**Figure 6:**
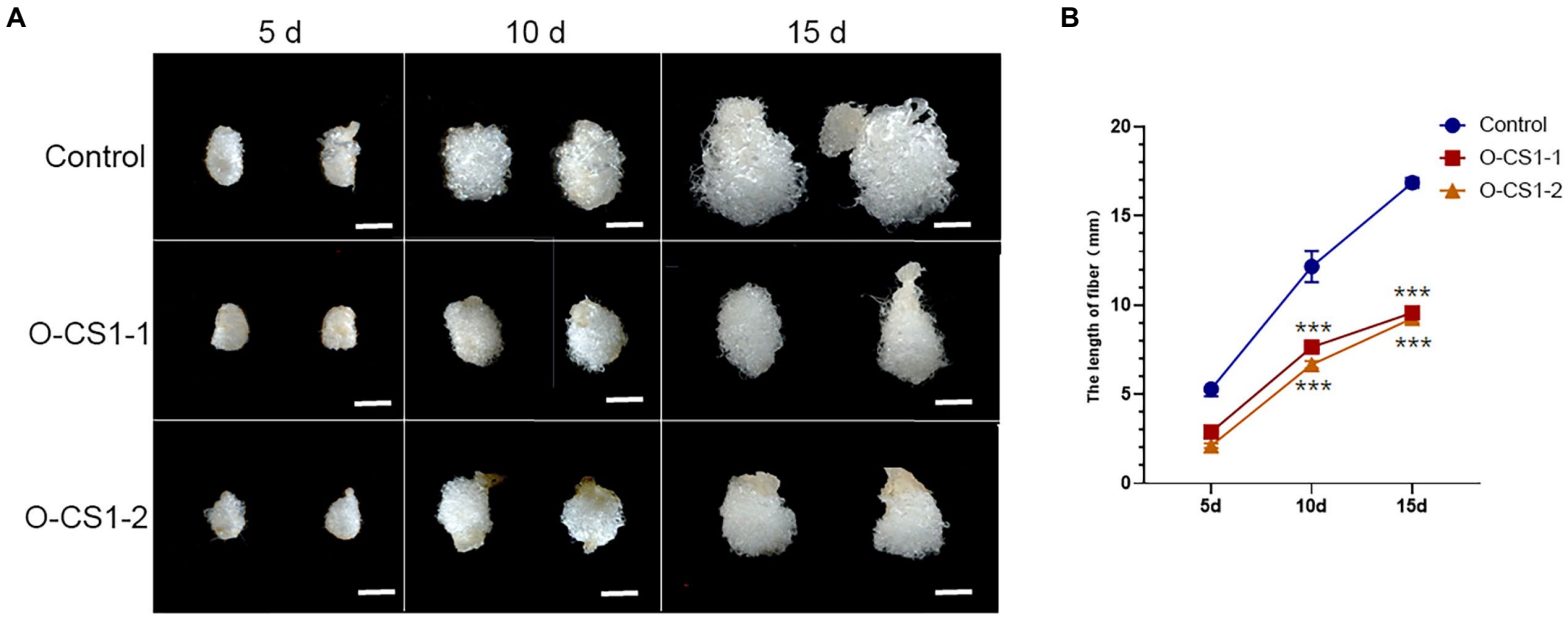
Cotton fiber growth in the ovule culture system *in vitro*. **(A)** The ovules of control and transgenic cotton plants grew in the ovule culture system *in vitro*; **(B)** The fiber length of cultured fiber cells *in vitro*. Control: Wild-type cotton; O-CS1-1and O-CS1-2: The transgenic cotton line 1# and 2# overexpressing *GhCS1* cotton. Data underwent a Student’s *t*-test: ^***^ indicate *p* < 0.001, respectively, vs. the wild-type. Bar = 4 mm.

Given that *GhCS1*–overexpression lines are highly sterile, we then examined the effect of overexpression of *GhCS1* on fiber elongation by *in vitro* ovule culture system. On the day of flowering, the transgenic flowers and control flowers were artificially pollinated with wild-type pollen and labeled. The 1-DPA ovules of O-*GhCS1*-1, O-*GhCS1*-2, and control were put into BT medium for dark culture at 32°C, and their fiber growth was observed and their length was measured. The results showed that the fiber lengths of O-*GhCS1*-1and O-*GhCS1*-2 were obviously shorter than those of the wild type after 5-, 10-, and 15-day cultures (Figures 6A,B), indicating that overexpression of *GhCS1* gene could severely inhibit fiber elongation.

### Sphingolipid profile of *GhCS1-*overexpression lines

**Figure 7:**
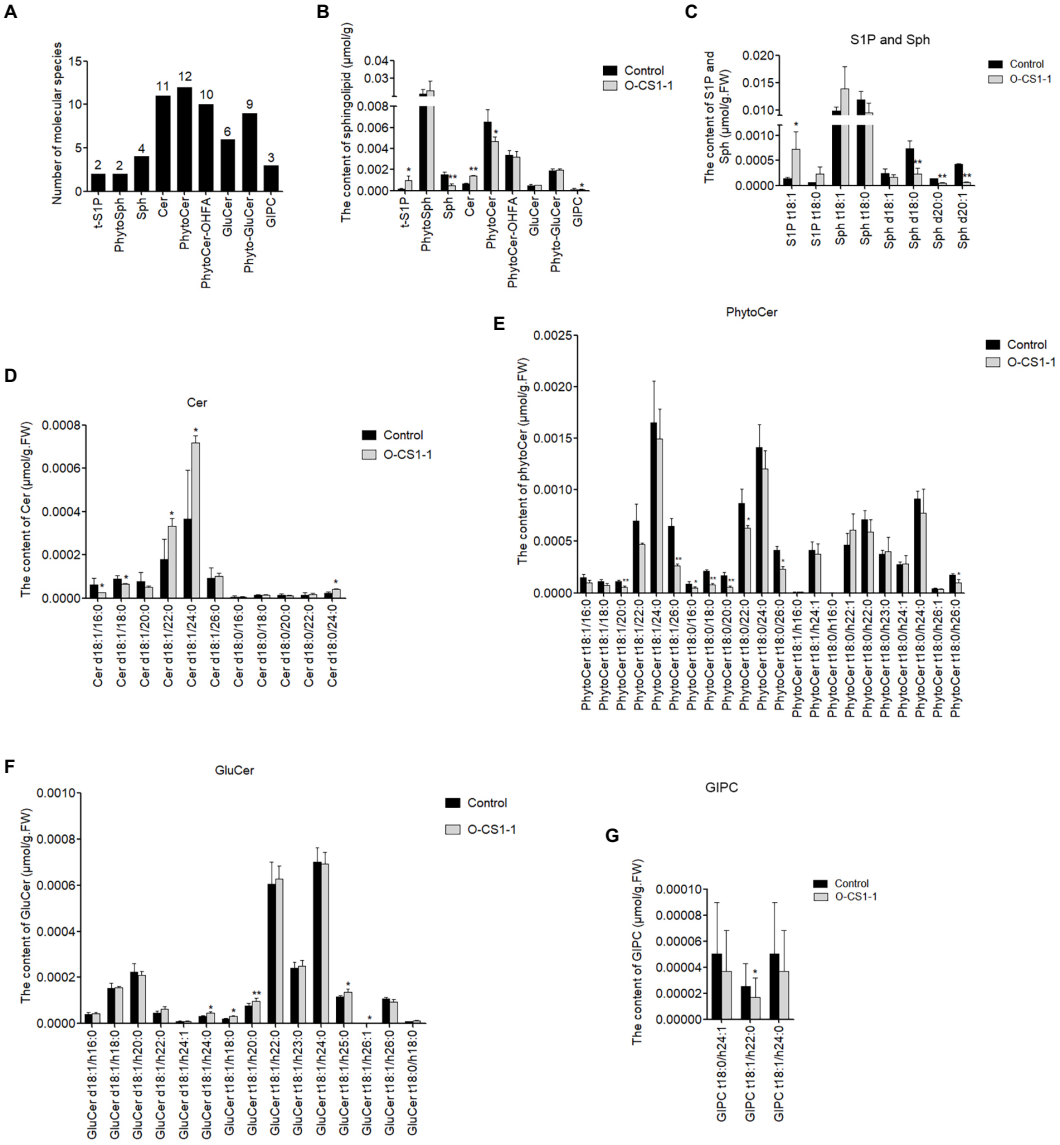
The difference of sphingolipid between control and transgenic 0-DPA ovule (with fiber cell). **(A)** Nine classes of sphingolipid detected and the number of each class in 0**-**DPA ovule. **(B)** The total concentration of each class of sphingolipid in control and transgenic ovule (with fiber cell). **(C–G)** The types and contents of various sphingolipid molecules in control and transgenic ovules (with fiber cell). Cer: ceramides; GIPC: Glycosyl Inositol Phospho Ceramides; GluCer: glucosylceramides; Phyto-GluCer: phyto-glucosylceramides; Phyto-Cer: phyto-ceramides; Phyto-Cer-OHFA: Phyto-ceramides with hydroxylated fatty acyls; Phyto-Sph: phyto-sphingosines; Sph:sphingosines; t-S1P: phyto-S1P. Data underwent a Student’s *t*-test: ^*^ and ^**^ indicate *p* < 0.05 and *p* < 0.01, respectively, vs. the control.

To understand the effect of overexpressing *GhCS1* on the composition and content of sphingolipids in cotton ovules and fibers, we detected the changes of sphingolipids in 0-DPA transgenic ovules (with fiber cells). The results showed that nine classes of sphingolipids were detected in 0-DPA ovules, which were phyto-sphingosine-1-phosphate (t-S1P), phyto-sphingosine (Phyto-Sph), sphingosine (Sph), ceramide (Cer), phyto-ceramide (Phyto-ceramide), hydroxylated fatty acyl phyto-ceramide (Phyto-Cer-OHFA), glucosylceramide (GluCer), phyto-glucosylceramide (Phyto-GluCer) and glucose inositol phosphate ceramide (GIPC). Each class contains 2, 2, 4, 11, 12, 10, 6, 9, and 3 molecular species, respectively (Figure 7A). Among them, there are 33 types of ceramide molecules, followed by GluCer, with 15 kinds of molecules.

Compared with the control, the content of Sph in 0-DPA ovules of transgenic cotton decreased significantly, while the content of Phyto-Sph (trihydroxysph) had no change significantly, and the content of phyto-S1P (t-S1P)increased significantly (Figure 7B). Considering that both Sph and Phyto-Sph are substrates of ceramide synthase, this result suggested that GhCS1 preferentially used Sph as substrates to synthesize ceramide. The content of ceramide increased significantly while the Phyto-Cer obviously decreased, further indicating that GhCS1 preferentially used Sph to synthesize ceramide (Figure 7B). Phyto-Cer-OHFA, GluCer, and Phyto-GluCer exhibited no difference in the 0-DPA ovules between control and transgenic cotton. Although GIPC was significantly reduced in transgenic 0-DPA ovules, the total content of GIPC was very low (Figure 7B). These results showed that overexpression of *GhCS1* gene promoted the synthesis of ceramide in cotton ovules, proving that *ChCS1* has the function of ceramide synthase. Furthermore, the GhCS1 preferentially used Sph as substrate.

Additionally, two t-S1P molecules were detected in 0-DPA ovules and the content of two molecules increased in transgenic ovules, whereas two phyto-Sph molecules (t18:1and t18:0) were detected and there was no significant difference between transgenic and control ovules. Four Sph molecules (d18:1, d18:0, d20:1, and d20:0) were detected and all of them were sharply reduced in transgenic cotton ovules (Figure 7C), indicating that they might be the dominant substrate of GhCS1. A total of 11 Cer molecules were detected, and the content of Cer molecules that containing VLCFA chains (C22, C24, and C26) increased significantly in transgenic cotton ovules, especially d18:1/22:0, d18:1/24:0, and d18:0/24 increased more than twice (Figure 7D), indicating that the dominant FA substrate of GhCS1 is VLCFA. A total of 22 Phyto-Cer molecules were detected, among which the content of 8 Phyto-Cer molecules (Phyto t18:0/16:0, Phyto t18:0/18:0, Phyto t18:0/20:0, Phyto t18:0/26:0, Phyto t18:1/20:0, Phyto t18:1/26:0, Phyto t18:0/h26:0, and Phyto t18:0/h26:0) significantly decreased in transgenic cotton ovules (Figure 7E). A total of 15 GluCer molecules and 3 GIPC molecules were detected, among which the content of 5 GluCer molecules increased significantly in transgenic cotton ovules (Figure 7F) and the content of 1 GIPC molecule decreased significantly in transgenic cotton ovules (Figure 7G). These results showed that overexpression of *GhCS1* gene significantly changed the composition and content of sphingolipids in transgenic cotton ovules, revealing that the dominant substrates of GhCS1 might be Sph and VLCFA. Given that the dominant substrates of AtLOH1 and AtLOH3 in *Arabidopsis* are Phyto-Sph and VLCFA, while the dominant substrates of AtLOH2 are Sph and LCFA. It is speculated that the function of GhCS1 is different from that of three ceramide synthetases in *Arabidopsis*.

## Discussion

### *GhCS1* may be a new ceramide synthase with special substrate preference

Ceramide is the center of sphingolipid biosynthesis and catabolism. It is also a key bioactive lipid that mediates or regulates a variety of cellular reactions. Therefore, enzymes involved in ceramide synthesis and catabolism play important roles in plant development. At the same time, these enzymes also have complex characteristics and functions (Mullen et al., 2012). So far, at least 33 different enzymes are identified to be involved in the synthesis and catabolism of ceramide (Futerman, 2019). There are six ceramide syntheses (ceramide synthase1 ~ 6, CerS1 ~ CerS6) in mammals and three ceramide synthetases LONGEVITY ASSURANCE GENE ONE HOMOLOG (LOH), AtLOH1 (At3g25540), AtLOH2 (At3g19260), and AtLOH3 (At1g19260) in *Arabidopsis* (Markham et al., 2011; Ternes et al., 2011). These ceramide synthases have different substrate specificity (Kim et al., 2021). CerS1mainly uses C18-CoA as a substrate to synthesize ceramide (Venkataraman et al., 2002), CerS2 mainly uses VLCFA(C22 to C24; Laviad et al., 2008), CerS3 uses far longer VLCFA(≥C26-CoA; Mizutani et al., 2006), CerS4 mainly uses C18-and C20-CoA (Riebeling et al., 2003), CerS5 and CerS6 mainly uses C16-CoA (Mizutani et al., 2005). In addition, CerSs also showed stereo-specificity for the long-chain base (LCB; Venkataraman and Futerman, 2001). *AtLOH1* and *AtLOH3* of *Arabidopsis* mainly use phyto-LCB and VLCFA (>C20), while *AtLOH2* mainly uses LCB and LCFA (Luttgeharm et al., 2015). In this study, we found that there are 10 CS homologous genes in upland cotton genome. Based on the phylogenetic tree analysis, these 10 CS were divided into three groups. The group I, which is closely related to *AtLOH1* and *AtLOH3* in the phylogenetic relationship, included four genes, Gh_D13G0934, Gh_A13G2275, Gh_A12G2623, and Gh_D12G1577. The group III, which is associated with *AtLOH2* in phylogenetic relationship, included four genes, Gh_A12G0215、Gh_D13G0393、Gh_D12G0217, and Gh_A13G0350. The group II, including two genes, Gh_D07G0583 and Gh_A07G0513, was an independent group with similar phylogenetic relationship to the group I and group III. Compared with three CSs in *Arabidopsis*, the sequence homology of GhCS1 with AtLOH1, AtLOH2, and AtLOH3 is 57.5%, 44.7%, and 60.3%, respectively, while the homology of AtLOH2 with AtLOH1 and AtLOH3 is 44.0% and 47.3%, respectively. These results suggested that *GhCS1* might be a new gene with a specific sequence. Generally, a gene function mainly depends on the gene sequence. Through analyzing the sphingolipid profile in 0-DPA ovules (with fiber cells) of control plants and overexpressed *GhCS1* transgenic plants, we found that the content of Sph (di-hydroxyl sphingosine) decreased significantly while the content of Phyto-Sph (tri-hydroxyl sphingosine) did not change significantly. The content of ceramide (containing Sph) increased significantly while the content of Phyto-ceramide (containing a Phyto-Sph) decreased significantly (Figure 7B). These results indicated that *GhCS1* was not only a functional ceramide synthase, but also preferentially used Sph as a substrate to synthesize ceramide. On the other hand, based on the content changes of various molecular species of ceramide, we found that the content of ceramide molecules containing VLCFA (C22, C24, and C26) increased significantly in transgenic cotton ovules when compared with control. The result indicated that GhCS1 preferentially used VLCFA as a substrate to synthesize ceramide. Taken together, GhCS1 preferentially used Sph and VLCFA as substrates to synthesize ceramide, which was different from AtLOH1, AtLOH2, and AtLOH3. AtLOH1 and AtLOH3 mainly catalyzed the tri-hydroxyl Sph and VLCFA to condensate ceramide, while AtLOH2 mainly catalyzed the condensation of di-hydroxyl Sph and LCFA (such as C16 fatty acyl-coenzyme A; Luttgeharm et al., 2015) Furthermore, overexpressing *GhCS1* inhibited cotton plant growth, which was similar to overexpressing *AtLOH2* in *Arabidopsis* (Luttgeharm et al., 2015). Therefore, *GhCS1* might be a new ceramide synthase with a special substrate preference.

### The composition and content of sphingolipids play some roles in cotton fiber development

In upland cotton, two *lintless-fizzless* mutants, *Xuzhou 142* and *Xinxiang Xiaoji*, are damaged in fiber cell initiation. Compared with the 0-DPA ovules of wild type, the GluCer and GIPC were significantly reduced in the 0-DPA ovules of mutants, while four ceramide molecules (d18:1/22:0 and d18:1/24:0, t18:0/22:0, and t18:0/h22:1) were significantly increased in mutants, indicating that the synthesis and catabolism of sphingolipids in mutant 0-DPA ovules were disordered. The decrease and increase of the content of these sphingolipids may be closely related to the initiation changes of fibers (Wang et al., 2021b). In this study, overexpressing *GhCS1* gene inhibited fiber cell initiation and the contents of four ceramide molecules (d18:1/22:0, d18:1/24:0, d18:1/26:0, and d18:0/24:0) were significantly increased in transgenic 0-DPA ovules. Particularly, the most abundance ceramide molecules (d18:1/22:0, d18:1/24:0) in 0-DPA ovules significantly increased in *GhCS1* transgenic ovules and two *lintless-fuzzless* mutant ovules. It was suggested that these two ceramide molecules might play an important role in the fiber cell initiation. On the other hand, exogenous application of the inhibitor of sphingolipid synthesis (such as FB1 or myriocin) seriously suppressed fiber cell elongation in the culture system of cotton ovule *in vitro* (Wang et al., 2020, 2021b; Chen et al., 2021). Furthermore, by analyzing the difference in sphingolipid composition and content between fiber cell elongation stage and secondary cell wall deposition stage, we demonstrated that the phyto-ceramide containing hydroxylated and saturated VLCFA (such as Phyto-Cer t18:1/h26:0 and Phyto-Cer t18:0/h26:0) was important for fiber cell elongation (Chen et al., 2021). However, this kind of ceramide molecules declined in the *GhCS1* transgenic ovules (with fibers), which might be a cause of inhibiting fiber cell elongation.

It is speculated that there are two reasons for the decrease of Phyto-Ceramide content. On the one hand, the increase of *GhCS1* activity inhibits the activity of CSs that uses Phyto-Sph and VLCFA as preferential substrates through an unknown mechanism, thus suppressing the synthesis of Phyto-Ceramide. On the other hand, since *GhCS1* uses Sph and VLCFA as preferential substrates, *GhCS1* activity increase consumed more VLCFA, so it is difficult to synthesize Phyto-Ceramide by other CSs. However, the conjectures need to be proved by further experiments.

### The balance between various sphingolipid components might be important for fiber cell development

In the ovules overexpressing *GhCS1*, not only the downstream products of ceramide synthase (such as Cer and Phyto-Cer) changed significantly, but also upstream intermediates (such as t-S1P, Sph) also greatly altered. Ceramide, Sph, or S1P are regarded as signal molecules that regulate plant development and stress response. It was reported that Sph and Cer induce the hypersensitive response (HR) or programmed cell death (PCD) in plants, whereas their phosphorylated forms show the opposite effect (Liang et al., 2003; Shi et al., 2007; Alden et al., 2011; Lachaud et al., 2011). Furthermore, S1P has been shown to affect stomatal aperture *via* the abscisic acid (ABA)-mediated signaling pathway, and S1P signaling may be closely related to G protein-coupled signaling pathways (Coursol et al., 2003). In this study, t-S1P and Cer increased while Sph and Phyto-Cer decreased in transgenic ovules, and the fiber cell initiation and elongation were inhibited. It was suggested that the balance between various components of sphingolipid might be important for fiber cell development.

## Data availability statement

The original contributions presented in the study are included in the article/Supplementary material, further inquiries can be directed to the corresponding author.

## Author contributions

GL and ML: conceptualization. QW and GW: methodology. QM: software. ML, FX, and GL validation. ML: formal analysis and project administration. QW: investigation. QC and YH: data curation. GL and ML: writing-original draft preparation. QW, ML, and FX: writing-review and editing. FL: visualization. ML and FX: supervision. ML and QW: funding acquisition. All authors contributed to the article and approved the submitted version.

## Funding

This research was funded by the National Natural Science Foundation of China, grant nos. 31971984 and 31571722, the Chongqing graduate scientific research innovation project, grant no. CYS21138, and the Genetically Modified Organisms Breeding Major Project of China, grant no. 2018ZX0800921B.

## Conflict of interest

The authors declare that the research was conducted in the absence of any commercial or financial relationships that could be construed as a potential conflict of interest.

## Publisher’s note

All claims expressed in this article are solely those of the authors and do not necessarily represent those of their affiliated organizations, or those of the publisher, the editors and the reviewers. Any product that may be evaluated in this article, or claim that may be made by its manufacturer, is not guaranteed or endorsed by the publisher.

## Footnotes

^1^ http://www.lipidall.com/

^2^ http://www.cottonfgd.org/
