# Supplementary material for: Overexpression of a ceramide synthase gene,GhCS1, inhibits fiber cell initiation and elongation by promoting the synthesis of ceramides containing dihydroxy LCB and VLCFA
Source: Front Plant Sci. 2022 Sep 2;13:1000348. doi: 10.3389/fpls.2022.1000348 (PMC9478514; doi:10.3389/fpls.2022.1000348)
Supplement: Supplementary file 1 [file Table_3.DOCX]

Table S1. Primers used for QRT-PCR and PCR analyses

| gene | Forward (5'-3') | Reverse (5'-3') |
| --- | --- | --- |
| GhCS1 | CTCAACCACACCTCATTTCAC | TTTCGTCACCGTTCGTGTGT |
| GhHis | GAAGCCTCATCGATACCGTC | CTACCACTACCATCATGGC |
| GhCS1::eYFP | CTCAACCACACCTCATTTCAC | CATCCTCATGTTCATCCTCGCCTTC |
| GhCS1 RT | GGGATTTTAAGGATGATGTTGATG | TCCAATTGATCCAGGGTGAAG |
| 35S | ACGACAGGACACACCCTCTTG |  |
| NOS | GGATCTGAGCTACACATGCTC |  |
