# Supplementary figures and images for: Overexpression of a ceramide synthase gene,GhCS1, inhibits fiber cell initiation and elongation by promoting the synthesis of ceramides containing dihydroxy LCB and VLCFA
Source: Front Plant Sci. 2022 Sep 2;13:1000348. doi: 10.3389/fpls.2022.1000348 (PMC9478514; doi:10.3389/fpls.2022.1000348)

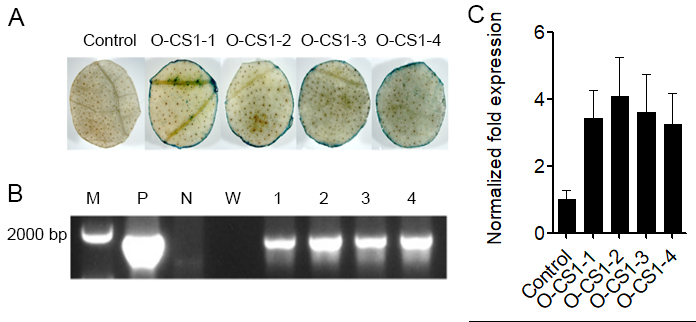

Supplement: Supplementary file 2 [file Image_1.JPEG]
